# Aggressive angiomyxoma: The first case report in skull

**DOI:** 10.3389/fsurg.2022.985739

**Published:** 2022-08-17

**Authors:** Zexin Cao, Lifeng Miao, Min Liu, Wenyu Liu, Hengrui Zhang, Xuchen Liu, Jiwei Wang, Xinyu Wang

**Affiliations:** ^1^ Department of Neurosurgery, Qilu Hospital, Cheeloo College of Medicine, Shandong University and Institute of Brain and Brain-Inspired Science, Shandong University, Jinan, China; ^2^ Key Laboratory of Brain Function Remodeling, Qilu Hospital of Shandong University, Jinan, China; ^3^ Department of Neurosuregery, Qilu Hospital of Shandong University Dezhou Hospital (Dezhou People’s Hospital), Dezhou, China; ^4^ Operating Room of Qilu Hospital, Shandong University, Jinan, China

**Keywords:** case report, aggressive angiomyxoma, local invasion, recurrence, skull

## Abstract

Aggressive angiomyxoma (AAM) is a rare mesenchymal tumor primarily growing in the soft tissue of the pelvis and perineum in women of reproductive age. It is a benign tumor that still has a probability of being accompanied by localized invasion. Although negative margins of resection are difficult to achieve due to the invasive nature of the tumor and the lack of a well-defined capsule, the first line of treatment for AAM is surgery. The diagnosis of AAM is difficult to make due to a lack of specific manifestations and specific tumor markers. In this study, we reported a case of aggressive angiomyxoma in a 2-year-old girl that rarely develops in the skull with craniocerebral compression. The patient initially had a mass on her head that attracted the attention of her family, and then she began to have episodic headaches. Surgery was performed after hospitalization, and the tumor recurred 1 year after the operation, around the originally affected skull.

## Introduction

Steeper and Rosai, for the first time, reported on aggressive angiomyxoma (AAM), which is a rare mesenchymal tumor primarily arising in the soft tissue of the pelvis and perineum in women of reproductive age, in 1983 (1). Male patients with AAM are less common, with a male-to-female ratio of 6.6:1. The peak incidence occurs in the fourth decade of life, but the tumor can appear any time between the ages of 9 months and 82 years (2). Although most cases are benign, these tumors are often locally invasive and have a high recurrence rate. Due to the rarity of this tumor, misdiagnosis is a common problem (3). To date, >400 cases have been reported in the literature, and although described as an indolent tumor, three cases of metastatic disease have been documented (4). This tumor is very rare and occurs mostly in the soft tissue of the perineum, almost never been found in the head. According to our search, no case reports of cranial AAM have been identified. We therefore report a novel case of a craniocerebral space–occupying lesion in a 2-year-old girl. After the operation, the pathological results showed an aggressive angiomyxoma of skull. One year later, the patient presented with similar symptoms. The patient was re-hospitalized for surgery, and the postoperative pathological results showed that it was consistent with the recurrence of AAM. This rare case fully demonstrates the invasiveness and easy recurrence of aggressive angiomyxoma, and because AAM so rarely occurs in the head, this report provides crucial clinical guidance for the diagnosis and treatment of brain space-occupying lesions in the future.

## Case report

**Figure 1:**
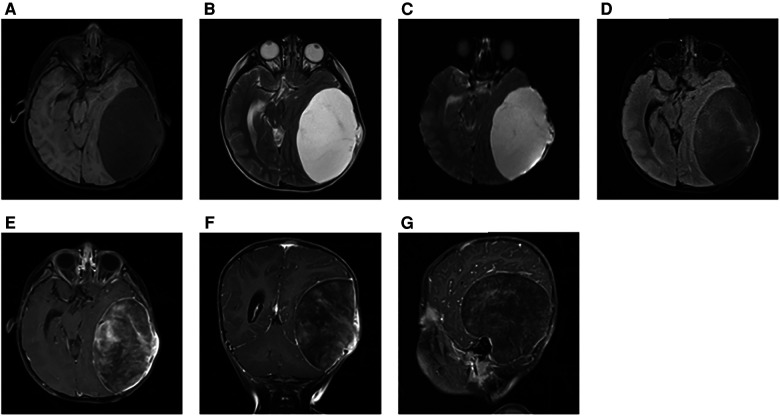
At the first illness, on T1-weighted and T2-weighted images, the tumor revealed long signal abnormal signals (**A,B**). In FLAIR, it was an iso-low signal (**C**). DWI showed a low-signal tumor (**D**). Contrast-enhanced MRI images showed a markedly enhanced mass (**E–G**).

**Figure 2:**
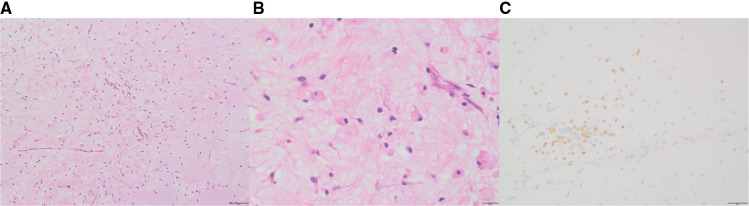
After the first surgery, hematoxylin and eosin staining of aggressive angiomyxoma (AAM): short spindle cells are seen on a mucous background, showing invasive growth (**A**:10×, **B**: 40×); in immunohistochemistry, S-100 was positive (**C**).

**Figure 3:**
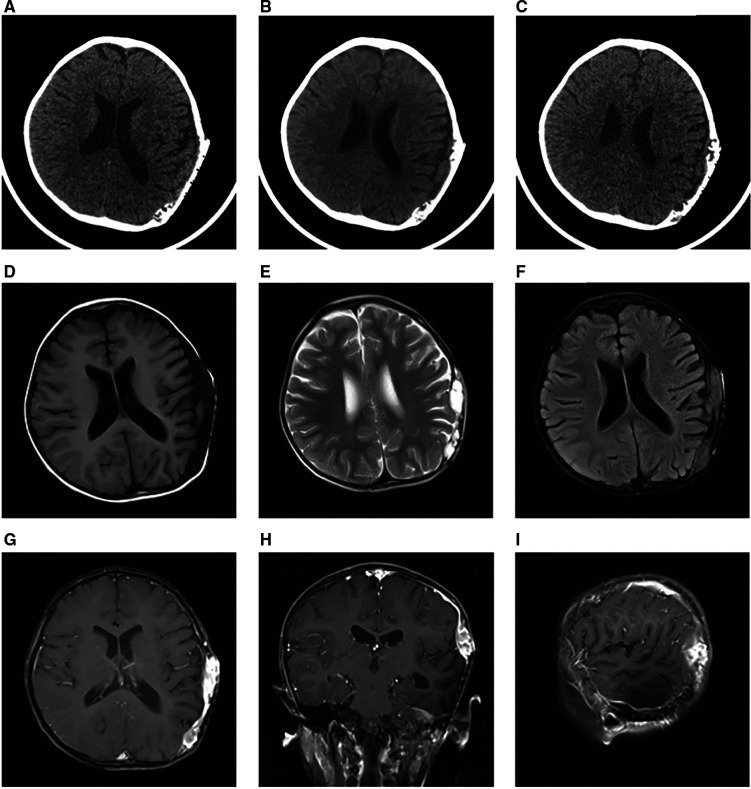
One year after the first operation, the CT scan showed multiple cystic bone destructions around the original cranial repair site (**A–C**). Around the repaired skull, the tumor revealed long signal abnormal signals on T1-weighted and T2-weighted images (**D,E**). In FLAIR, it was an iso-low signal (**F**). Contrast-enhanced MRI images also showed a markedly enhanced mass (**E–G**).

**Figure 4:**
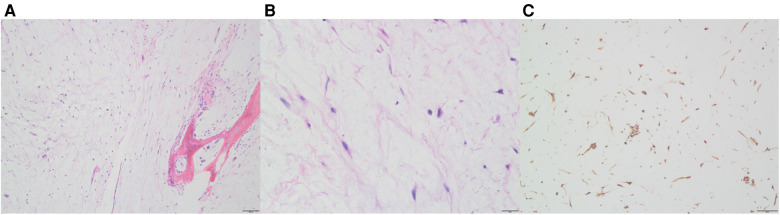
After the second operation, Hematoxylin-eosin (HE) staining and immunohistochemistry of the tumor. Thin-walled blood vessels surrounded by several spindle cells can be seen in the images (**A**: 10×, **B**: 40×); In immunohistochemistry, vimentin was positive (**C**).

Initially, the family of the 2-year-old girl in question accidentally discovered a mass behind the child's left ear, similar in size to an egg, without redness or swelling of the skin, and the child occasionally had symptoms of nausea and vomiting. At first, when observing these clinical presentations, we speculated it could be an osteoma in the skull of the child. Magnetic resonance imaging (MRI) examination showed a huge, cystic and oval in the left temporoparietal and posterior cranial fossa. On T1-weighted and T2-weighted images, the tumor revealed long signal abnormal signals (Figures 1A,B), an iso-low signal in FLAIR (Figure 1C), a flocculent slightly high signal in the inner part, and a slightly low signal and thin signal in the edge. And a slightly hypointense thin capsular shadow can be seen at the edge. Contrast-enhanced MRI images showed lamellar enhancement in the early stage after enhancement, and lamellar enhancement was visible in the capsule after delay, and the meninges were also significantly strengthened (Figures 1E-G). The lesions were hypointense on DWI, and were slightly isointense in patches (Figure 1D). Accompanied by skull destruction and absorption, the size of the mass was about 9.4 × 6.4 × 9.6 cm3. Imaging data showed disorganized internal structures and intracranial mass, which shattered our initial impression. Based on imaging, it was suspected that it was a meningioma, but the imaging features were not typical. The mass had an intact capsule and cystic structure, and it was difficult to make a clear diagnosis of the patient's disease based on her clinical symptoms and imaging data. Therefore, pathological diagnosis remains the gold standard. Due to intracranial compression and localized cranial invasion, we fully communicate with the patient's parents about the child's condition. Finally, we performed a left temporoparietal occipital craniotomy for the patient. During surgery, it was observed that the tumor was gray-yellow, located in the epidural layer, soft, with clear borders, containing mucus, and with invasion of the skull. The tumor was completely removed and a pathological examination revealed short spindle cells on a mucous background with mild cell morphology and aggressive growth (Figures 2A,B). The immunohistochemical results were cytokeratin pan (−), epithelial membrane antigen (EMA) (−), S100 (+), GFAP (−), and CD34 (−) (Figure 2C). Combined with the results of immunohistochemistry, two professors of pathology participated in the diagnosis, and finally the tumor was considered to be aggressive angiomyxoma. We then developed a reasonable review plan for the patient. About 1 year after the operation, left craniocerebral space–occupying postoperative findings were observed during a routine CT scan of the repaired multiple cystic bone destruction of the surrounding skull, and the MRI data also revealed that there was aberrant space occupied near the cranioplasty with abnormal signals of long T1 and long T2, an iso-low signal in FLAIR, a flocculent slightly high signal in the inner part, and a slightly low signal and thin signal in the edge. Meanwhile, a slightly hypointense thin capsular shadow can be seen at the edge (Figure 3). Combined with the patient's medical history and imaging findings and the diagnosis of two pathology professors, it was concluded that the patient had AAM recurrence, so further surgery, named Left-sided craniotomy for brain tumor and lesion craniectomy, was arranged and properly expanding the scope of surgical resection. During the operation, it was found that the tumor tissue adhered under the skull bone flap around the repair was gray-yellow and soft, with a clear boundary. The tumor tissue was covered with mucus, and we completely removed the tumor tissue. Pathological manifestations were as follows: gray-white, gray-red broken tissue, with a total volume of 9 cm × 7 cm × 2 cm, most of which was bone. The tumor contains small thin-walled blood vessels, and proliferating spindle cells can be seen around the vessels (Figures 4A,B). With combined immunohistochemical analysis [EMA (−), vimentin (+), desmin (−), S100 (±), CD34 (−), cytokeratin pan (−), ER (−), PR (−)] and medical history-taking, we considered a recurrence of aggressive angiomyxoma (Figure 4C). We then advised the child's parents to bring the pathology report to the oncology department for further treatment, and we requested regular follow-up CT or MRI scans. Imaging data from 2020 to 2021 revealed no evidence of a recurrence, indicating that appropriate expansion of surgical resection, supplemented by appropriate radiotherapy and chemotherapy, could improve the prognosis of AAM patients (**Supplementary Figures**).

## Discussion

AAM is a rare mesenchymal tumor. In women, it usually occurs in the perineum and deep pelvic soft tissues, while, in men, it usually occurs in the groin–scrotal region. Due to disease specificity and clinical rarity, misdiagnosis occurs in ≥80% of cases (5, 6). According to our search, Zhu et al. reported a case of AAM of maxilla ineffective to radiotherapy (7), and Lin et al. found a case of AAM of the male buttock (8). Additionally, Rivard et al. observed an AAM that had grown in the vulva and bladder (9), while Agarwal et al. reported an AAM of the renal pelvis in a horseshoe-shaped kidney (10). In addition, there are some AAMs that grow in other parts, but we have not yet retrieved literature reports of AAMs in the head.

The AAM discussed in this case occurred in the left temporo-parietal occipital region and the posterior fossa with erosion of the skull. The incidence of this disease is low, and it is generally more common in the reproductive area of women of childbearing age. When we first came into contact with this case, we therefore hardly considered AAM. At initial onset, there were no positive signs in the nervous system, and the headache could have been related to the intracranial mass effect of the tumor. MRI revealed no obvious features of myxoma, and we often classify such cases as craniocerebral space–occupying lesions of unknown nature in the preoperative diagnosis. During the operation, we found that the tumor was a gray-yellow tumor with a volume of 9 cm × 8.5 cm × 5 cm. It was gray-yellow, hard, and translucent, and the surface was sticky and slippery. The size of the violated skull area was 7.5 cm × 5.5 cm, and a pathological diagnosis was made to determine the tumor's nature. Due to the rarity of such tumors and the patient's non-specific symptoms, early diagnosis required a high degree of suspicion (11). Such lesions can grow continuously and have local invasiveness, and they will have a mass effect on the closed space of the skull, even affecting the function of the brain functional area or invading the skull. Recurrence is also a major feature of this disease, no matter what the growth site is. Although it is benign, the term “aggressive” is used due to the high rate of local recurrence after resection (12, 13). When we encounter space-occupying lesions of unknown nature, we should be alerted to the occurrence of AAM so as not to delay treatment. Surgery is the current first-line treatment, but radical resection does not significantly reduce recurrence compared to limited resection (14). Pathological diagnosis is the current gold standard for diagnosis (11).

The pathogenesis of this tumor has not been elucidated. At present, chromosomal translocations involving 12q13-15 have been reported in a variety of mesenchymal neoplasms, including lipomas, liposarcomas, leiomyomas, and pulmonary hamartomas (15). A number of reports of a variety of benign mesenchymal tumors have involved chromosomal translocation of 12q13-15, and *HMGA2* has been identified as a target gene. *HMGA2*, a member of the high mobility–group protein family, is a structural transcription factor expressed primarily during embryogenesis, with low expression detected in normal adult tissues, whereas the expression of *HMGA2* is not detected in terminally differentiated cells (16, 17). Cytogenetic changes in the *HMGA2* gene have been identified in various stromal tumors, such as lipomas and leiomyomas, and partially studied in AAM. Further genetic analysis of AAMs could elucidate which are the most common molecular events involving *HMGA2* (e.g., breaks within the third intron or outside *HMGA2*) and whether these distinct molecular mechanisms have clinical behavioral relevance, such as suggesting the likelihood of relapse (18).

AAMs are usually unencapsulated, with poorly defined borders, and can blend in with surrounding soft tissue. The tumors vary in size from 1 to 60 cm and are often tan, bulky, and rubbery with a glossy, gel-like surface (11). Histologically, AAMs consist of a myxoid matrix with mixed collagen fibers and dilated thick-walled vessels (19). Microscopically, the tumor consists of bland spindle cells, loosely arranged in a myxoid edematous stroma, with vascular proliferation of various sizes (20). In the histological analysis of the present case, short spindle cells were seen on a mucous background with mild, aggressive growth of the cells, combined with previous cases of perineal AAM that demonstrated a spindle cell neoplasm with numerous blood vessels in a myxoid background, highly suggestive of AAM (21–23). At the same time, we also need to conduct immunohistochemical analysis. AAM may be positive for desmin, vimentin, CD34, and estrogen and progesterone receptors and negative for S100 protein; however, these findings could vary between cases (24). In this case of craniocerebral AAM, S100 protein was positive and desmin, vimentin, CD34, and estrogen and progesterone receptors were negative at the initial stage of illness. The lesion only occurred in the skull at the time of recurrence, and its immunohistochemical analysis revealed that only vimentin was positive. The first-line treatment for AAM is surgery, although negative resection margins are difficult to achieve due to the infiltrative nature of the tumor and the absence of a defined capsule (25). However, in many cases, due to the particularity of the tumor growth site or the complexity of the growth location, or in order to preserve certain functions, it is difficult to achieve complete resection. At this time, partial resection is acceptable, but follow-up observation is required. Zhu et al. conducted a retrospective analysis of 27 AAM cases and found a recurrence rate of 37%, and in their study found that the surgical margin was an independent factor affecting PFS (26). Nowadays, hormone therapy, vascular embolization and radiation therapy have gradually started to be applied, which can effectively reduce the recurrence rate of the disease. In this case, although a complete surgical resection was performed at the first onset, the patient recurred after a lapse of 1 year. The recurrence location was similar to the original location, but only the skull was affected. Therefore, regular follow-up after surgery is very necessary.

Chemotherapy and radiation therapy have no clear roles in the treatment of AAM, as the tumor has a low mitotic index. We had recommended the patient visit the oncology department for consultation, but we subsequently lost her to follow-up and were unable to assess the clinical guidance of chemoradiotherapy. Because the patient was both ER-/PR-negative and young, we did not recommend hormone therapy. According to previous reports, the prognosis of AAM is generally good. AAM tumor metastasis is rare, but the rate of local recurrence for AAM is approximately 30%–40%, and recurrence could occur 10–15 years after initial resection (27). Li et al. evaluated the clinical presentations, treatments, and follow-ups of two patients with AAM in the scrotum in their hospital, and among 34 cases reported in the literature, they found that recurrence occurred in three cases (9.09%) at the primary sites and no cases of distant metastasis (28). However, Stella et al. presented a case of aggressive angiomyxoma in a young woman with multiple local recurrences that metastasized to the lungs, killing the patient (29). For this reason, long-term follow-up with MRI or CT scans is recommended (30).

In conclusion, AAM is a rare mesodermal tumor, and its clinical rarity determines that it is easy to misdiagnose. Moreover, the invasiveness and peripheral boundaries of AAM are unclear, indicating a high possibility of recurrence. Summarizing previous CT and MRI findings could give us clinical hints. Extensive surgical resection is the basis for radical AAM. We believe that the high recurrence rate is due to initial misdiagnosis and inadequate surgical resection. For residual or recurrent tumors, adjuvant therapy may be necessary. Features of hormone dependency suggest hormone therapy may be valuable (3). A reasonable review plan and adjuvant therapy after surgery could improve the overall prognosis of the tumor.

## Conclusion

Although aggressive angiomyxoma is primarily growing in the soft tissue of the pelvis and perineum in women of reproductive age. However, the incidence in different age groups and locations cannot be ignored. This case of AAM shows some features when he occurs in the head, including imaging, symptomology, etc., but it is still difficult to diagnose based on these alone. Surgery remains the first choice of treatment, postoperative histopathological examination is still the gold standard for its diagnosis.

## Data Availability

The original contributions presented in the study are included in the article/**Supplementary material**, further inquiries can be directed to the corresponding author.
