# Supplementary material for: Aggressive angiomyxoma: The first case report in skull
Source: Front Surg. 2022 Aug 17;9:985739. doi: 10.3389/fsurg.2022.985739 (PMC9428339; doi:10.3389/fsurg.2022.985739)

Supplementary image

After the first operation

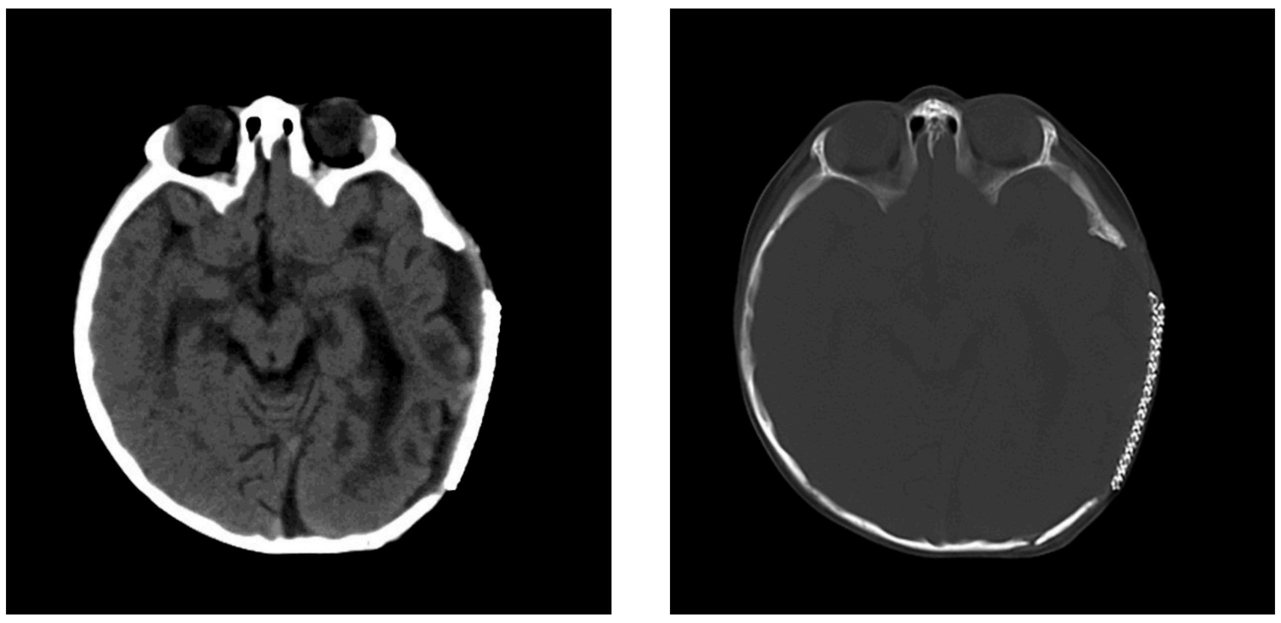

7 days, after the first operation

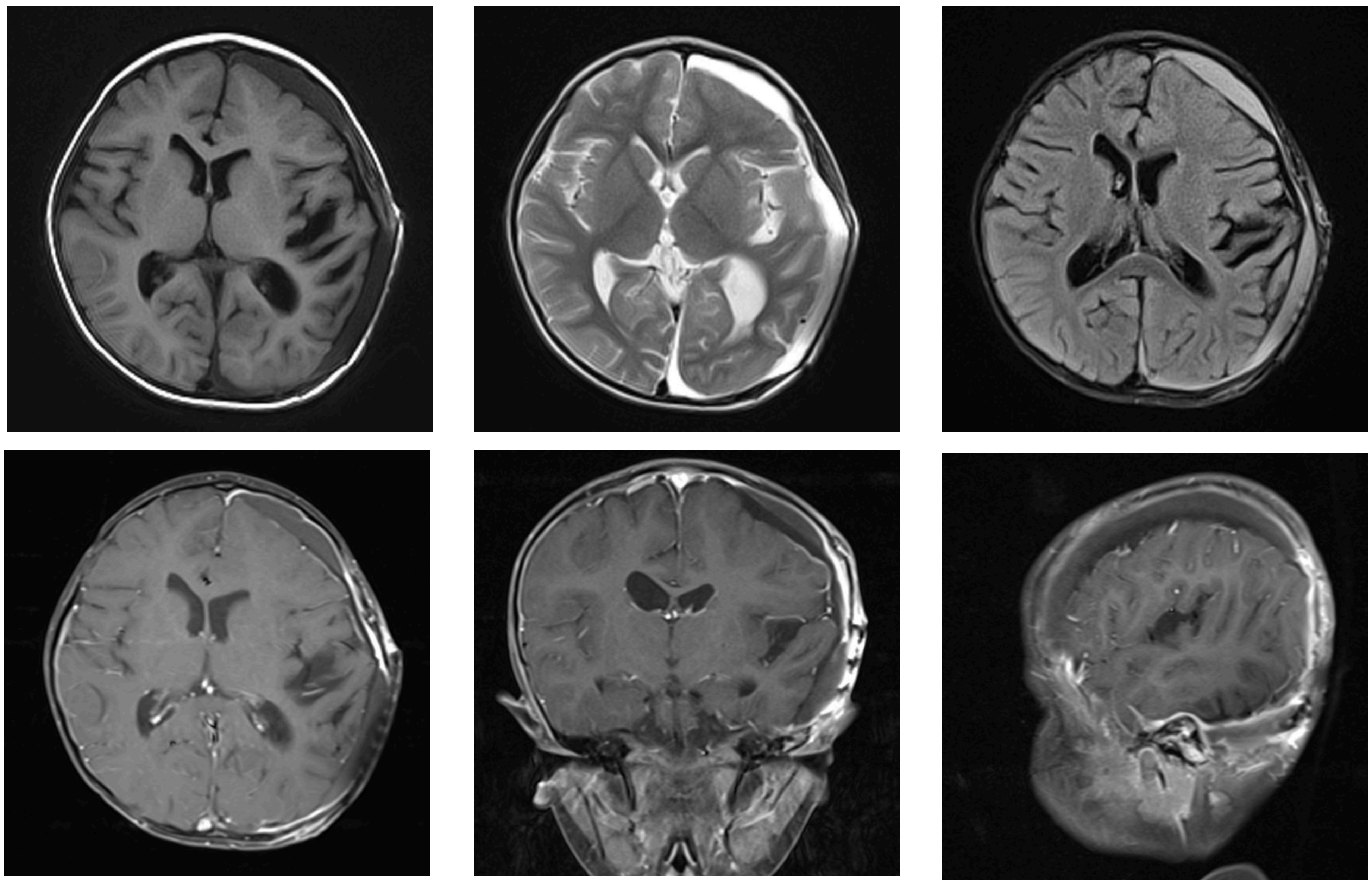

2 months, after the first operation

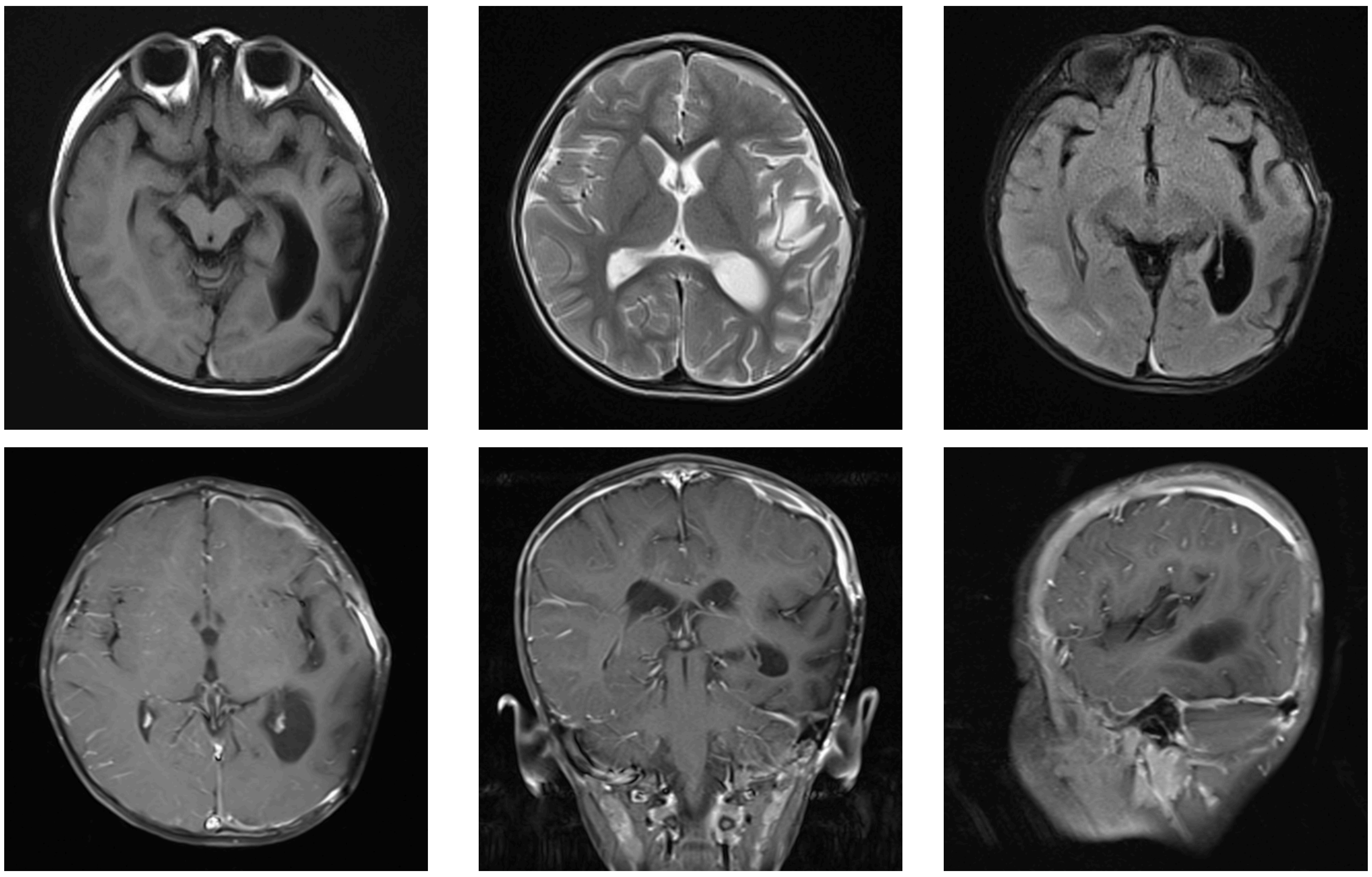

6 months, after the first operation

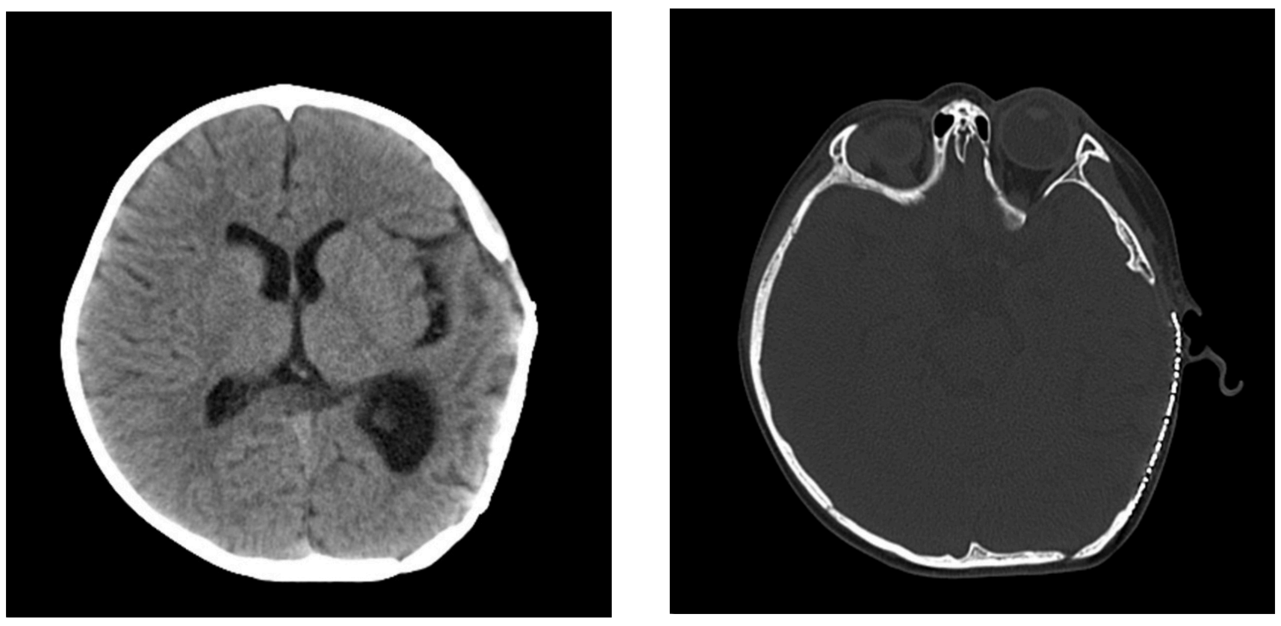

1 year, after the second operation

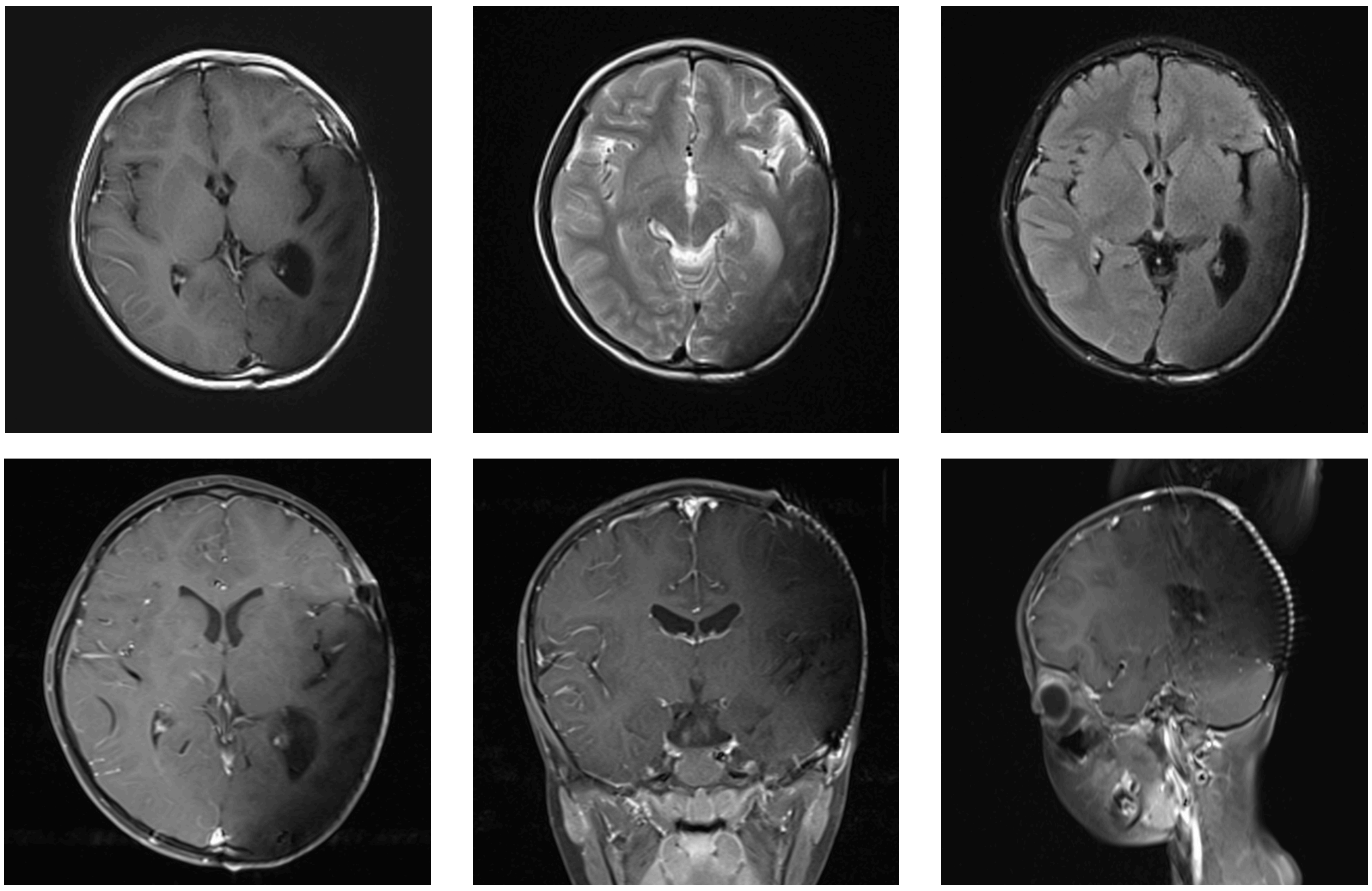

1 year, after the second operation

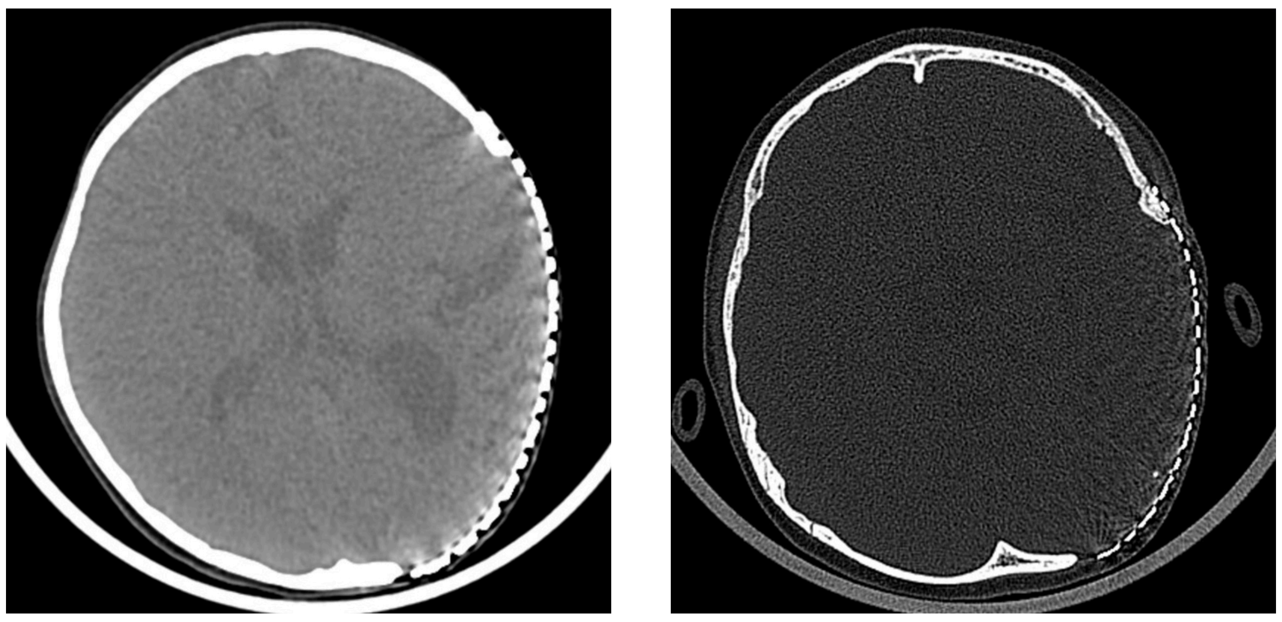

2 years, after the second operation

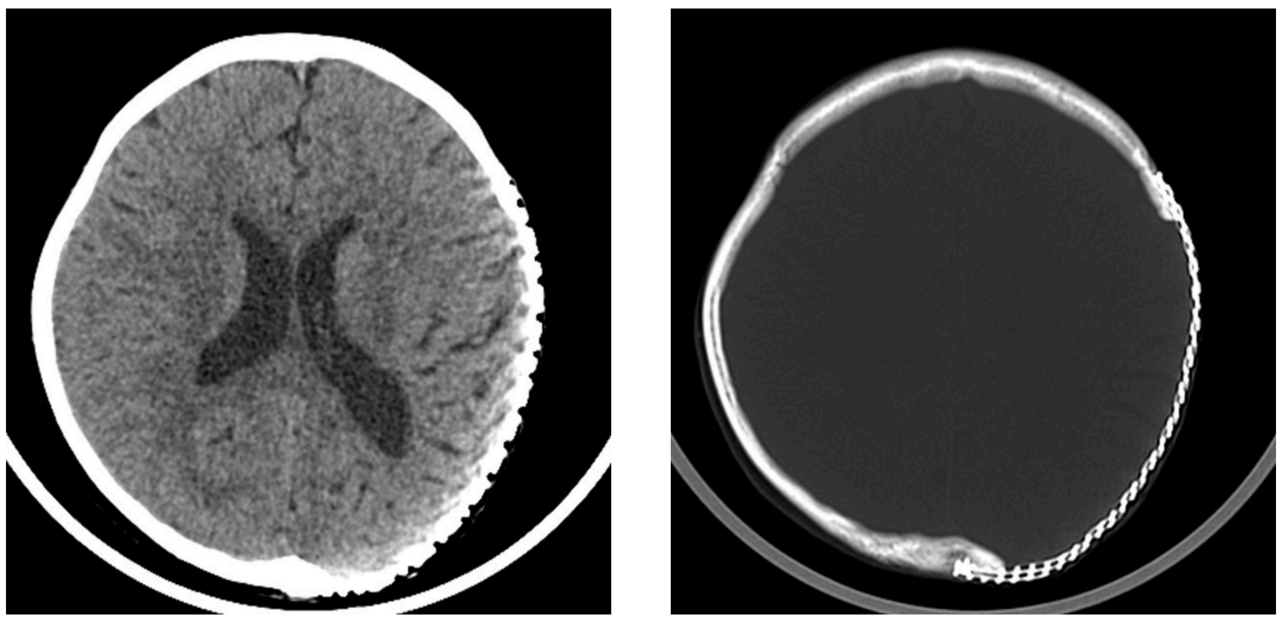

Supplement: Supplementary file 1 [file Data_Sheet_1_v1.pdf]
